# Increased Oral Bioavailability of Resveratrol by Its Encapsulation in Casein Nanoparticles

**DOI:** 10.3390/ijms19092816

**Published:** 2018-09-18

**Authors:** Rebeca Peñalva, Jorge Morales, Carlos J. González-Navarro, Eneko Larrañeta, Gemma Quincoces, Ivan Peñuelas, Juan M. Irache

**Affiliations:** 1 NANO-VAC Research Group, Department of Pharmacy and Pharmaceutical Technology, University of Navarra, 31008 Pamplona, Spain; rebe.penalva@gmail.com (R.P.); jmorales.3@alumni.unav.es (J.M.); e.larraneta@qub.ac.uk (E.L.); 2 Centre for Nutrition Research, University of Navarra, 31080 Pamplona, Spain; cgnavarro@unav.es; 3 Radiopharmacy Unit, Department of Nuclear Medicine, Clinica Universidad de Navarra, University of Navarra, 31008 Pamplona, Spain; gquinfer@unav.es (G.Q.); ipenuelas@unav.es (I.P.)

**Keywords:** resveratrol, casein, nanoparticles, oral delivery, mucus-permeating, bioavailability, resveratrol-*O*-3-glucuronide

## Abstract

Resveratrol is a naturally occurring polyphenol that provides several health benefits including cardioprotection and cancer prevention. However, its biological activity is limited by a poor bioavailability when taken orally. The aim of this work was to evaluate the capability of casein nanoparticles as oral carriers for resveratrol. Nanoparticles were prepared by a coacervation process, purified and dried by spray-drying. The mean size of nanoparticles was around 200 nm with a resveratrol payload close to 30 μg/mg nanoparticle. In vitro studies demonstrated that the resveratrol release from casein nanoparticles was not affected by the pH conditions and followed a zero-order kinetic. When nanoparticles were administered orally to rats, they remained within the gut, displaying an important capability to reach the intestinal epithelium. No evidence of nanoparticle “translocation” were observed. The resveratrol plasma levels were high and sustained for at least 8 h with a similar profile to that observed for the presence of the major metabolite in plasma. The oral bioavailability of resveratrol when loaded in casein nanoparticles was calculated to be 26.5%, 10 times higher than when the polyphenol was administered as oral solution. Finally, a good correlation between in vitro and in vivo data was observed.

## 1. Introduction

Resveratrol (Rsv) is a nutraceutical named *trans*-3,4′,5-trihydroxystilbene and consists of two aromatic rings which are linked through a methylene bridge. It is a natural polyphenol found in more than 70 species of plants, particularly in grapes, blueberries and peanuts [1]. One of the more important sources for human consumption is red wine, although its content depends on several factors including the grape variety, vineyard location, cultivation system, climate and soil type and production process among others [2]. The interest of the scientific community in resveratrol was originally sparked by epidemiological studies, that evidenced an inverse relationship between moderate wine consumption and cardiovascular disease (the so-called “French Paradox”) [3,4] and by the fact that cancer preventive properties of resveratrol was observed both in vitro and in vivo [5].

The mechanism by which resveratrol exerts such arrange of beneficial effects across species and disease models is not yet elucidated. However, these effects would be related to the capability of this polyphenol to enhance the nitric oxide bioactivity and/or its inhibitory effect on platelet COX-1 and NF-κB [6]. In this context, resveratrol would block the platelet aggregation by its ability to specifically inhibit prostaglandins synthesized by COX-1 [7] such as thromboxane A2, which is a potent inducer of platelet aggregation and vasoconstrictor [8]. Apart from the vasorelaxant effect due to the inhibition of thromboxane A2 synthesis, resveratrol would also induce vasodilatation by its ability to enhance nitric oxide signaling in the endothelium [9], leading to a reduction in its inactivation rate. On the other hand, it has been demonstrated that resveratrol possesses cancer chemopreventive and chemotherapeutic activity [5,10]. This fact would be related with its inhibitory effect over cyclooxygenase [2], NF-κB [11] and protein kinase C [12], among others. Moreover, resveratrol would inhibit the tumor-induced neovascularization acting as an antiangiogenic agent [10,13].

Despite all these advantages, resveratrol also possesses some drawbacks that limit its use. Thus, resveratrol shows a very low aqueous solubility (0.02–0.03 mg/mL [14]. In addition, resveratrol is very sensitive to degradation by exposure to oxygen, light, temperature and oxidative enzymes [6]. Under these circumstances *trans*-resveratrol may be isomerized to the less active *cis*-version, reducing its activity [15,16]. Another limiting and important factor is its poor oral bioavailability (less than 5%) due, at least in part, to its rapid metabolism and elimination [17]. Resveratrol suffers a presystemic metabolism through first-pass glucuronidation and sulfate conjugation of the phenolic groups in the intestinal enterocytes and liver. In addition, resveratrol hydrogenation of the aliphatic trans double bond by the intestinal microbiota was shown. The major resveratrol metabolites encountered after oral administration of resveratrol includes resveratrol-3-*O*-sulfate, resveratrol-3-*O*-glucuronide and dihydroresveratrol conjugates which results in low oral bioavailability [6,18]. Very little is known about the bioactivity of these metabolites; although resveratrol-3-*O*-sulfate would show a strong in vitro anti-estrogenic activity [19].

The aim of this work was to prepare, characterize and evaluate the capability of casein nanoparticles as carriers for the oral delivery of resveratrol. Casein is a food-grade protein that may be transformed into nanoparticles under mild conditions [20] and, thus, produce devices with interest for food fortification as well as for nutraceutical and pharmaceutical applications. In addition, milk caseins display a high affinity for polyphenols, including resveratrol [21,22], and can bind these types of molecules via hydrophilic and hydrophobic interactions. Furthermore, in the presence of proteins, resveratrol would be more protected from *trans*-to-*cis* isomerization than when it is in the free form [23,24]. More particularly, this work reports the capability of these devices to promote the oral bioavailability of resveratrol in rats.

## 2. Results

### 2.1. Characterization of Nanoparticles

Table 1 shows the physicochemical characteristics of casein nanoparticles used in this study. For empty nanoparticles, the mean size was around 140 nm and the zeta potential was negative. When resveratrol was encapsulated into casein nanoparticles, the resulting average size significantly increased (210 nm vs. 140 nm) whereas the negative zeta potential was slightly more negative than for empty nanoparticles (−19 mV vs. −12 mV). The resveratrol loading was calculated to be 31 µg/mg nanoparticles, with an encapsulation efficiency close to 70%.

**Figure 1:**
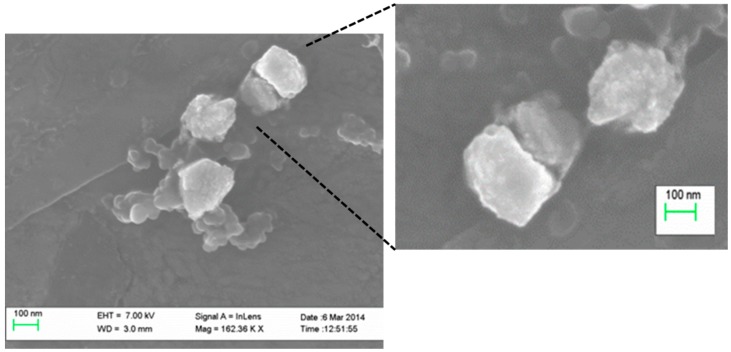
Scanning Electron Microscopy microphotographs obtained from resveratrol-loaded casein nanoparticles.

The morphological analysis by field emission scanning electron microscopy (Figure 1) showed that resveratrol-loaded casein nanoparticles consisted of homogeneous populations of polyhedral nanoparticles with rough surface and apparent size similar to that obtained by photon correlation spectroscopy.

### 2.2. In Vitro Release Studies

**Figure 2:**
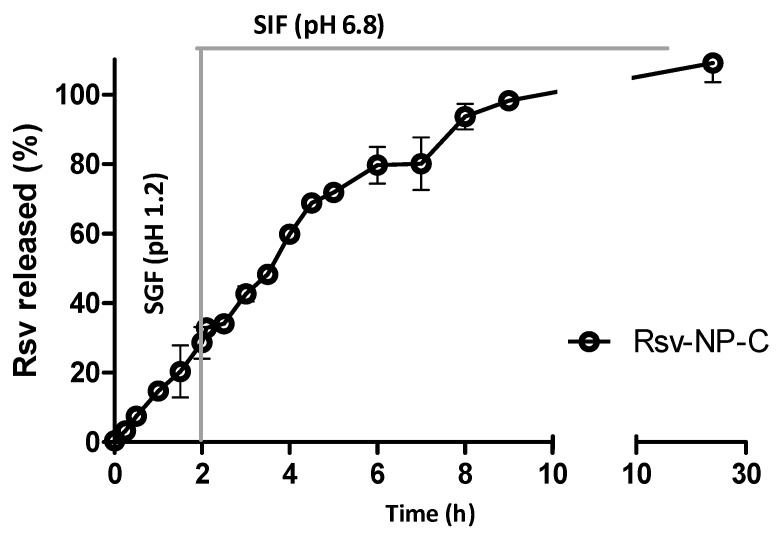
Resveratrol release profile from casein nanoparticles after incubation in simulated gastric (SGI, pH 1.2; 0–2 h) and simulated intestinal fluids (SIF, pH 6.8; 2–24 h) under sink conditions. Data expressed as mean ± SD, *n* = 3.

Resveratrol release kinetics from casein nanoparticles were evaluated in simulated physiological fluids. For this purpose, nanoparticles were initially incubated during the first 2 h in simulated gastric fluid (SGF) and, then, in simulated intestinal fluid (SIF). Figure 2 represents the release profile of resveratrol from casein nanoparticles as the cumulative percentage of the cargo released versus time. Interestingly, the release of resveratrol from casein nanoparticles was found to be independent of the pH conditions. During the first 2 h, under SGF (pH 1.2), about 30% of the loaded resveratrol was released. Then, four hours later (in SIF) the amount released was close to 80% of the total content of resveratrol. Nine hours after the beginning of the experiment, almost the total amount of the encapsulated resveratrol was released from nanoparticles.

The resveratrol release data from casein nanoparticles fitted well to both the Korsmeyer-Peppas model (K_KT_ = 0.14 ± 0.01 h^−*n*^; *n* = 1.02 ± 0.04; *R*^2^ > 0.99) and the zero-order kinetics equation (K = 0.14 ± 0.00 h^−1^; *R*^2^ > 0.99). These results suggest that the release mechanism of resveratrol from casein nanoparticles would be ruled by the erosion of the nanoparticulate matrix.

### 2.3. In Vivo Biodistribution Study of Casein Nanoparticles

**Figure 3:**
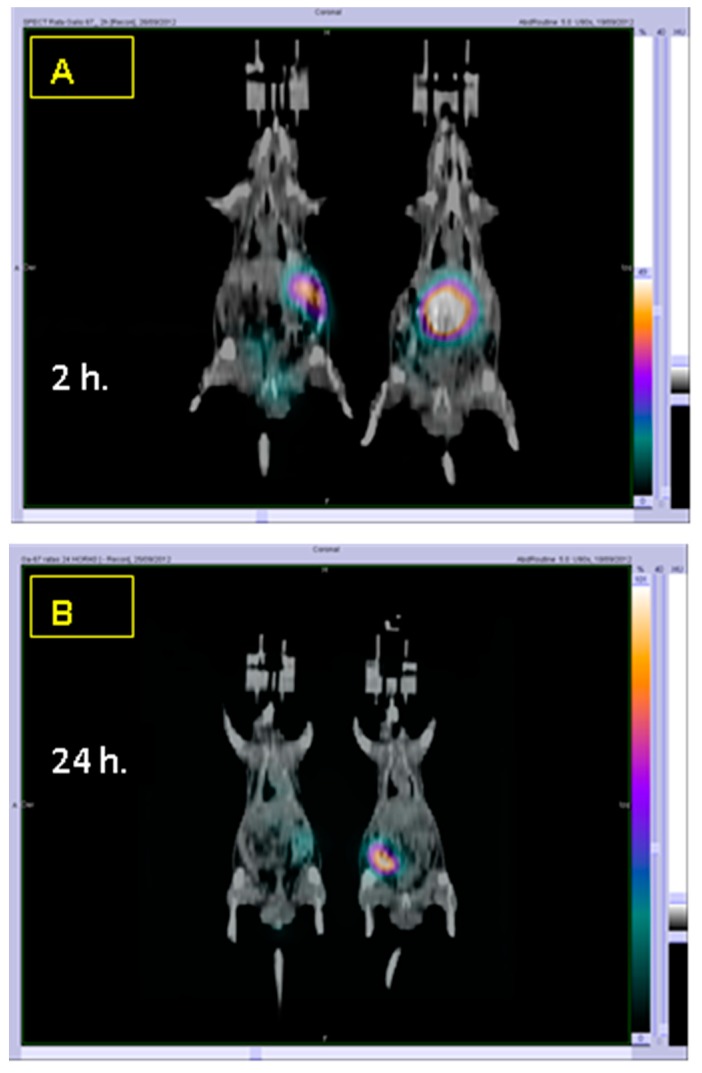
Comparison of the biodistribution of casein nanoparticles radiolabeled with gallium (NP-NOTA-Ga) and the radiomarker used as control (Gallium-67 citrate). Panels (**A**) and (**B**) show gammacamera images after oral administration of NP-NOTA-Ga (rat on the right) and control (rat on the left) at 2 and 24 h post administration.

Figure 3 shows the biodistribution of casein nanoparticles, at different times after their oral administration to rats. Two hours after their oral administration, casein nanoparticles were visualized in the stomach. Twenty-two hours later, the radioactivity was found in the distal part of gastrointestinal tract; whereas, 2 days post administration, the radioactivity had disappeared. For the control formulation of gallium-67, during the 48-h in which the experiment took place, the radiolabeled agent (gallium-67 citrate) was always visualized in the stomach. This finding would be related with the ready formation of Ga(OH)_3_ and GaO(OH), that possess a very low aqueous solubility over a wide pH range [25].

**Figure 4:**
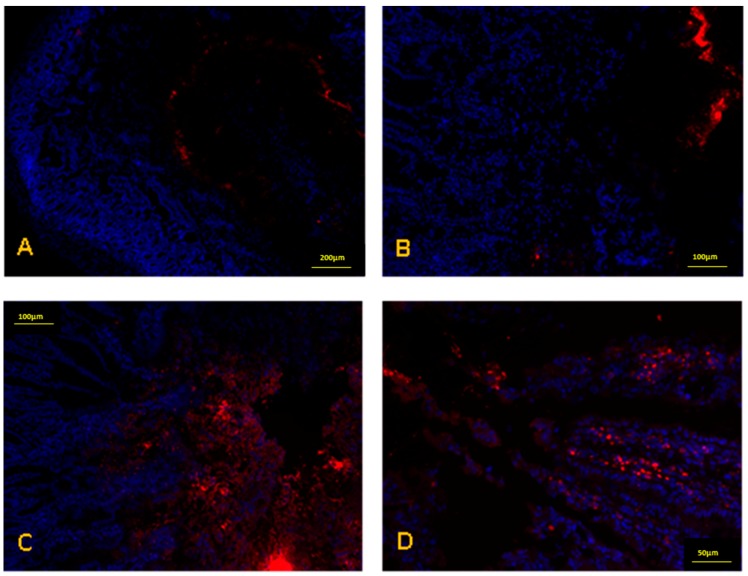
Fluorescence microscopy images of rat jejunum samples, two hours after the oral administration of either an aqueous suspension of Lumogen^®^ red (**A**,**B**) or casein nanoparticles labelled with Lumogen^®^ red (**C**,**D**). Nuclei of cells were stained blue with DAPI (4′,6-diamidino-2-phenylindole).

Figure 4 shows fluorescence microscopy images of jejunum samples, 2 h after the oral administration to animals of fluorescently labelled nanoparticles. The control, an aqueous suspension of Lumogen^®^ red, was visualized as agglomerates in the intestinal lumen of animals (Figure 4A,B). On the contrary, casein nanoparticles were observed within the mucus layer and in close contact with the surface of the epithelium (Figure 4C,D).

### 2.4. Pharmacokinetic Studies in Wistar Rats

**Figure 5:**
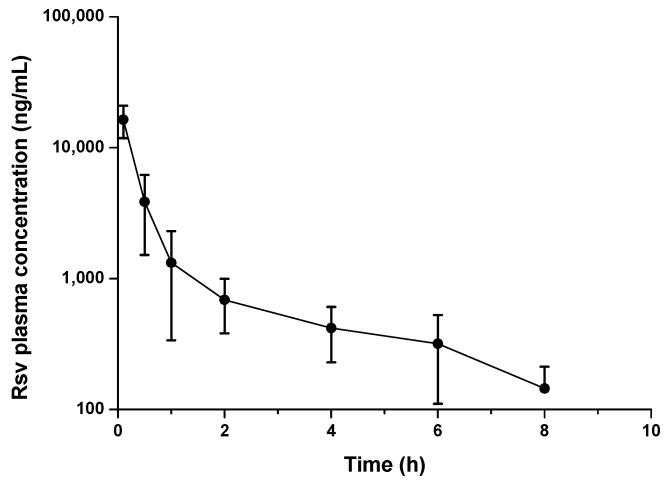
Resveratrol plasma concentration vs. time after a single i.v. administration of a resveratrol solution (dose 15 mg/kg). Data expressed as mean ± SD, (*n* = 6).

The plasma concentration-time profile of a resveratrol solution (in a mixture of PEG-400 and water; 1:1 by vol.) after its intravenous administration of a single dose (15 mg/kg) to rats is shown in Figure 5. Data were adjusted to a non-compartmental model. As it can be seen, the resveratrol plasma concentration decreased rapidly in a biphasic way, and no quantifiable levels were detected 8 h post administration. The peak plasma concentration (C_max_) of resveratrol was around 15 µg/mL. Values obtained for AUC (Area Under the Curve) and half-life (t_1/2_) were 11.4 µg h/mL and 2.0 h, respectively. The resveratrol clearance and its distribution volume were calculated to be 0.2 L/h and 0.6 L, respectively (Table 2).

**Figure 6:**
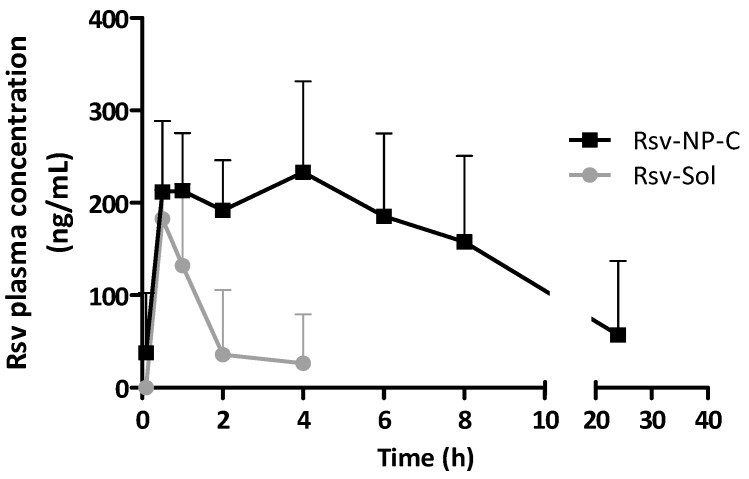
Resveratrol plasma concentration vs. time after a single oral administration of 15 mg/kg for the different formulations tested. (i) Resveratrol PEG400:water solution (●); (ii) Resveratrol-loaded casein nanoparticles (■). Data expressed as mean ± SD, (*n* = 6).

Figure 6 shows the plasma concentration levels of resveratrol as a function of time after a single oral administration of 15 mg/kg to male Wistar rats of the different formulations tested (solution, suspension and casein nanoparticles). When the aqueous suspension of resveratrol was administered orally, no quantifiable levels of the polyphenol were detected in the plasma of laboratory animals. For the solution of resveratrol (Rsv-Sol) orally administered, the polyphenol plasma levels were only quantifiable during the first 4 h post administration. Interestingly, when resveratrol was loaded in casein nanoparticles, the polyphenol plasma levels were quantified during 24 h. In addition, at least during the first 8 h, casein nanoparticles provided sustained levels of resveratrol in plasma.

Pharmacokinetic parameters of resveratrol in plasma, after its intravenous or oral administration, are listed in Table 2. When resveratrol was administrated as a PEG400:water solution by the oral route, the AUC was around 0.29 µg h/mL. In the case of resveratrol encapsulated in casein nanoparticles, the AUC was found to be significantly higher than for the conventional formulation (*p* < 0.01). In addition, both MRT and t_1/2_ values of resveratrol in plasma after the administration of the nanoparticles was found to be around 6-fold higher than when resveratrol was administered as oral solution. On the other hand, the volume of distribution of the polyphenol as well as the resveratrol clearance and t_1/2_ when administered in casein nanoparticles were similar to the values calculated after its administration by the intravenous route. Finally, the relative oral bioavailability of resveratrol when incorporated into nanoparticles achieves 27%, about 10-times higher than the value obtained when the polyphenol was formulated as oral solution.

**Figure 7:**
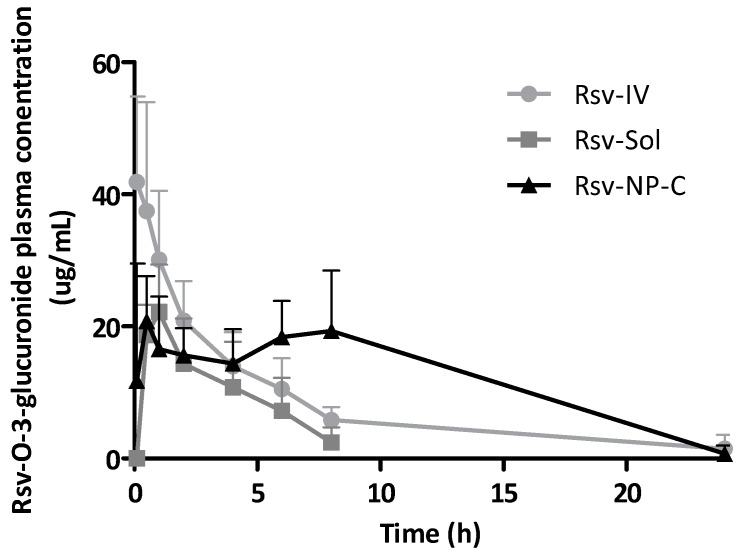
Resveratrol-*O*-3-glucuronide plasma concentration vs time after the administration of a single dose of resveratrol (15 mg/kg) in casein nanoparticles (oral) or dissolved in a PEG400:water mixture (oral and intravenously). (i) Resveratrol solution intravenously administered (Rsv-IV) (●); (ii) resveratrol solution orally administered (Rsv-Sol) (■); (iii) resveratrol-loaded casein nanoparticles orally administered (Rsv-NP-C) (▲). Data expressed as mean ± SD, (*n* = 6).

Figure 7 shows the concentration of one of the main metabolites of resveratrol (resveratrol-*O*-3-glucuronide) in plasma as a function of time, after the administration of a single dose of resveratrol encapsulated in nanoparticles (oral) or dissolved in a PEG400:water solution (oral and intravenous). As expected, the metabolite plasma levels were higher than those observed for the polyphenol (Figure 6). However, the profiles of the curves were similar for both compounds, irrespective of the route of administration.

When resveratrol was administered intravenously, its metabolite concentration reached 42 µg/mL (C_max_) and, then, its levels decreased rapidly reaching a very low amount in plasma only 8 h post administration. The AUC value was calculated to be 197 µg h/mL. For the solution of resveratrol orally administered, the C_max_ of resveratrol-*O*-3-glucuronide in plasma was 2-times lower (22 µg/mL) than when administered intravenously. Moreover, the metabolite was only detected and quantified during the first 8 h after administration. The AUC value was calculated to be 104 µg h/mL.

Concerning Rsv-NP-C, the profile of the curve representing the concentration of resveratrol-*O*-3-glucuronide in plasma vs time was characterized by increasing levels of the metabolite in plasma until reaching a plateau (around 20 µg/mL). Then, the plateau was maintained for at least 8 h. Twenty-four hours after the administration of casein nanoparticles, quantifiable but low levels of the resveratrol metabolite were found. The AUC of resveratrol-*O*-3-glucuronide, when the polyphenol was administered orally after its encapsulation in casein nanoparticles (295 µg h/mL), was found to be around 2-times higher than when the resveratrol was iv administered (*p* < 0.05) and 3-times higher than when the drug was orally administered as a PEG400:water solution (*p* < 0.01).

### 2.5. In Vitro-In Vivo Correlations

**Figure 8:**
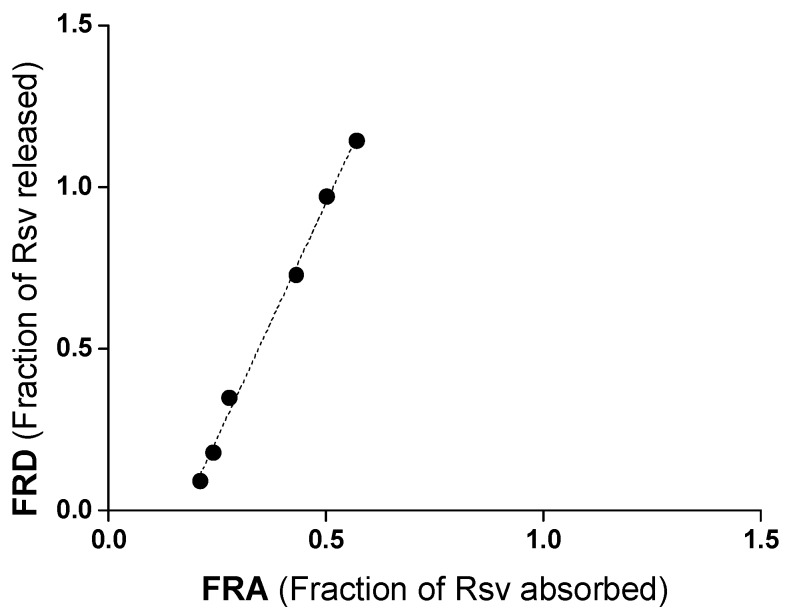
In vitro-in vivo correlation (IVIVC) plot for resveratrol when loaded in casein nanoparticles (Rsv-NP-C), showing fraction absorbed as a function of fraction released.

The relationship between the in vitro dissolution data and the in vivo pharmacokinetic data was examined by plotting the percentage of drug released until 8 h vs. the fraction of intact resveratrol absorbed during the same period (Figure 8). A good linear regression relationship was observed between the fraction of resveratrol released and the fraction of resveratrol absorbed (*R*^2^ = 0.996).

## 3. Discussion

This work reports the preparation and evaluation of casein nanoparticles as carriers for resveratrol. The use of casein may offer some advantages, including its capability to be used to produce nanoparticles as well as its ability to bind polyphenols in a non-specific way [21,22] and its protective effect against the inactivation of resveratrol by *trans*-to-*cis* isomerization [23,24].

The resulting resveratrol-loaded casein nanoparticles were polyhedral (Figure 1) and displayed a size of around 200 nm with resveratrol content close to 31 μg/mg nanoparticle. This result is at least similar to that reported by Jose and collaborators that used solid lipid nanoparticles for the delivery of this polyphenol [26]. In other recent works (using chitosan microspheres [27], lipid nanoparticles [28] or niosomes [29]), reported resveratrol loadings between 2- and 3-times lower than for the casein nanoparticles described here.

Interestingly, the release of resveratrol from casein nanoparticles was independent of the pH conditions. Moreover, the cumulative amount of resveratrol released from nanoparticles followed a zero-order kinetic (Figure 2). Thus, it is possible to speculate that the entry of water inside the casein nanoparticles would induce a relaxation and/or erosion of the nanoparticles matrix that would favor the resveratrol diffusion. A similar explanation was proposed in other works, in which they described the release of the biologically active compound as a result of the hydrolytic degradation of the sodium caseinate matrix through the entry of water inside the nanoparticles [30,31].

In the present study, the plasma concentrations of resveratrol provided by a conventional oral solution of the polyphenol in a mixture of PEG400 and water were low. With this solution, the oral bioavailability of resveratrol was calculated to be 2.6%. The highest recorded concentrations of resveratrol in plasma occurred 30 min after administration, and values returned to baseline within 4 h (Figure 6). These results are in line with the findings previously reported by other research groups [32,33,34]. This low oral bioavailability would be the result of the effect of UDP-glucuronosyltransferase and sulfotransferases on resveratrol. These enzymes, localized in the liver [35] and small intestine [17], would transform resveratrol in its glucuronides and sulfates derivatives.

On the other hand, casein nanoparticles provided higher and more prolonged resveratrol plasma levels than the oral solution. More important, the resveratrol plasma levels were sustained for at least 8 h post administration and significant amounts of the polyphenol was also quantified 24 h after the administration of nanoparticles to rats (Figure 6). Consequently, the relative oral bioavailability of resveratrol when administered after its encapsulation in casein nanoparticles was found to be 10-times higher than when formulated as oral solution (Table 2). This improvement in the relative oral bioavailability of resveratrol observed for casein nanoparticles was higher than other improvements reported in the literature. Thus, using SNEDDS (Self Nanoemulsifying Drug Delivery Systems), the resveratrol bioavailability was found to be 5-fold higher than the control formulation (an aqueous suspension of the polyphenol) [36]. In a similar way, solid lipid nanoparticles have also been proposed as carriers for improving the oral bioavailability of resveratrol [37]. In this particular case, these lipid nanoparticles offered an 8-times higher oral bioavailability than the control formulation (an aqueous suspension. In another interesting work, Ramalingam and Ko [38] defined a relative oral bioavailability of resveratrol of about 4-times higher from solid nanoparticles than that observed for a conventional suspension of the polyphenol.

Another important aspect is the effect of formulating resveratrol in casein nanoparticles on its primary pharmacokinetic parameters. Thus, with Rsv-NP-C, the values of the polyphenol half-life in plasma (t_1/2_), clearance (Cl) and volume of distribution (Vd) were similar to those obtained after its intravenous administration as solution. These findings would be evidence that nanoparticles remain within the gut of animals and just the released resveratrol was absorbed. This fact was confirmed by the biodistribution studies. Thus, radiolabeled casein nanoparticles, after their administration to rats, remained in the gastrointestinal tract and no proofs of nanoparticle “translocation” or absorption was observed (absence of signals in the liver, spleen, lungs or kidneys of animals; Figure 3). Furthermore, the fluorescently labelling of casein nanoparticles with Lumogen^®^ red allow us to visualize a fluorescent signal in the area in which the epithelium cells are located (Figure 4C,D). All of this suggests the mucus-permeating properties of casein nanoparticles and their capability to reach the surface of the epithelium in which the cargo (resveratrol) would be released. The release process would be drive by enzymatic digestion facilitating the erosion of the nanoparticle matrix and the elimination of nanoparticles. This would be in line with the work of Elzoghby and collaborators [39], in which they described a certain capability of casein nanoparticles to reach the surface of intestinal cells. After the release of resveratrol from casein nanoparticles, it would be absorbed through the enterocytes and, at least, a fraction would escape from its rapid enzymatic metabolization. In rats, the major metabolite of resveratrol is the glucuronide conjugate (resveratrol-*O*-3-glucuronide) [40] that, in our work, it was quantified in plasma. Interestingly, for the different formulations tested, the profile of the curves for the metabolite plasma levels (Figure 7) was similar to that observed for resveratrol (Figure 6). The highest AUC for the metabolite was found when resveratrol was administered encapsulated in casein nanoparticles (about 3-times higher than for Rsv-Sol). This fact demonstrated the controlled release properties of these carriers that can sustain the plasma concentration of the polyphenol for at least 8 h. Nevertheless, these elevated levels of the metabolite in plasma do not correlated adequately with a relatively high oral bioavailability observed with Rsv-NP-C. One possible explanation would be a saturation of the biotransformation phenomenon due to the presence of high local resveratrol concentrations. The saturation of glucuronosyltransferases, involved in the presystemic metabolism of resveratrol, by high levels of this polyphenol have been described previously [41,42]. Another possibility would be that the delivery of resveratrol via casein nanoparticles would facilitate to bypass the presystemic metabolism of this lipophilic compound by promoting its intestinal lymphatic transport. This possibility has been proposed for lipophilic compounds that may associate with lipoproteins, facilitating their entry to intestinal lymphatic vessels and, then, transported to systemic circulation [43].

Another important point to highlight is the good correlation found between the in vitro (resveratrol release from nanoparticles) and in vivo (resveratrol plasma levels) data (Figure 8). This finding would corroborate the capability of casein nanoparticles to carry resveratrol until the surface of the absorptive membrane and, once there, to control its release at a rate that would favor its absorption and entry in the circulation.

## 4. Materials and Methods

### 4.1. Reagents

Sodium caseinate was purchased from ANVISA (Madrid, Spain). Resveratrol, lysine, mannitol, polyethylene glycol 400 (PEG 400), DAPI (4′,6-diamidino-2-phenylindole), formaldehyde and Tween 20, were obtained from Sigma-Aldrich (Darmstadt, Germany). Calcium chloride, ethanol, methanol, acetonitrile and acetic acid HLPC (High Liquid Performance Chromatography) grade were from Merck (Darmstadt, Germany). Resveratrol-3-*O*-d-glucuronide was from @rtMolecule (Cedex-France). Gallium-67 citrate from IBA Molecular SA (Pamplona, Spain) and NOTA from Macrocyclics Inc., (Dallas, TX, USA). Lumogen^®^ F red 305 was from Kremer (Aichstetten, Germany). Tissue-Tek^®^ OCT (Optimal Cutting Temperature) compound was obtained from Sakura (Alphen, The Netherlands). 4′,6-diamidino-2-phenylindole (DAPI) was obtained from Biotium Inc. (Madrid, Spain). All reagents and chemicals used were of analytical grade.

### 4.2. Preparation of Resveratrol-Loaded Nanoparticles

Resveratrol-loaded casein nanoparticles (Rsv-NP-C) were prepared as described previously [20] with some modifications. Briefly, 600 mg sodium caseinate and 60 mg lysine were dissolved in 40 mL purified water. In parallel, 26 mg resveratrol were dissolved in 2.6 mL ethanol and added to the casein solution. The mixture was incubated for 30 min under magnetic stirring at room temperature and the nanoparticles were formed by the addition of 24 mL of an aqueous solution of calcium chloride (0.8% *w*/*v*). The nanoparticles were purified by ultrafiltration (polysulfone membrane cartridge, 50 kDa; Medica SPA, Medolla, Italy) before adding an aqueous solution of mannitol as protectant (20 mL; 100 mg/mL). Finally, the suspensions were dried in a Mini Spray Drier B-290 apparatus (Büchi Labortechnik AG, Flawil, Switzerland) under the following experimental conditions: (i) inlet temperature of 90 °C; (ii) outlet temperature 45–50 °C; (iii) air pressure: 2–5 bar; (iv) pumping rate of 2–6 mL/min; (v) aspirator of 100% and (vi) air flow at 900 L/h.

Empty nanoparticles (NP-C) were prepared in the same way as described above but in the absence of resveratrol.

### 4.3. Preparation of Resveratrol Conventional Formulations

Two different formulations of resveratrol were also prepared. The first one consisted of a solution of the polyphenol in a mixture of PEG 400 and water (Rsv-Sol). For this purpose, 37.5 mg resveratrol were dissolved in 5 mL PEG 400 under magnetic stirring. Then 5 mL water were added and the mixture was maintained under agitation in the dark for 10 min.

The second one was an extemporary suspension of resveratrol (Rsv-Susp) in purified water. Briefly, 37.5 mg resveratrol were weighed and dispersed in 10 mL purified water under magnetic agitation for 10 min. The suspension was used after visual inspection for absence of aggregates (Size: 21.5 ± 9.24 µm; PDI: 0.510 ± 0.042).

### 4.4. Characterization of Resveratrol-Loaded Nanoparticles

#### 4.4.1. Physicochemical Characterization

The mean size and the zeta potential of nanoparticles were determined by photon correlation spectroscopy (PCS) and electrophoretic laser Doppler anemometry, respectively, using a Zetaplus apparatus (Brookhaven Instruments Corporation, Holtsville, NY, USA). The diameter of the nanoparticles was determined after dispersion in ultrapure water (1:10) and measured at 25 °C by dynamic light scattering angle of 90°. The zeta potential was determined as follows: 200 μL of the samples were diluted in 2 mL of a 0.1 mM KCl solution adjusted to pH 7.4.

The morphology of the nanoparticles was studied using a field emission scanning electron microscopy (FE-SEM) in a Zeiss DSM940 digital scanning electron microscope (Oberkochen, Germany) coupled with a digital image system (Point Electronic GmBh, Halle, Germany). The yield of the process was calculated by gravimetry [20].

#### 4.4.2. Resveratrol Analysis

Resveratrol was quantified by HPLC-UV [44]. Analysis were carried out in an Agilent model 1100 series LC and diode-array detector set at 306 nm. Data were analyzed in chemstation G2171 program (B.01.03). The chromatographic system was equipped with a reverse phase 150 mm × 2.1 mm C18 Alltima column (particle size 5 μm; Altech, Flemington, NJ, USA) and a Gemini C18 support AJO-7596 precolumn. The mobile phase was a mixture of water, methanol and acetic acid in a gradient condition and pumped at 0.25 mL/min. The column was heated at 40 °C and the injection volume was 10 µL. Under these conditions, the run time for resveratrol was 22.8 ± 0.5 min. Calibration curves in ethanol 75% were designed over the range of 1–100 µg/mL (*R*^2^ = 0.999).

For analysis, 10 mg nanoparticles were dispersed in 1 mL water before centrifugation at 17,000 rpm for 20 min at 4 °C, the supernatants were analyzed to determine the amount of free resveratrol (not encapsulated). The amount of resveratrol loaded in the nanoparticles was calculated by subtracting the amount of resveratrol quantified in the supernatant from the theoretical total amount of the polyphenol employed in the preparation of nanoparticles. Each sample was analyzed by triplicate and the results were expressed as the amount of resveratrol (in µg) per mg of nanoparticles. The encapsulation efficiency (*E.E.*) was calculated as follows:

(1) $$E.E.\;\left(\%\right)=\frac{\left(Rsv_{t}-Rsv_{s}\right)}{Rsv_{t}}\times 100$$

in which *Rsv_t_* is the total theoretical amount of resveratrol in the formulations and *Rsv*_s_ corresponds to the amount of resveratrol quantified in the supernatants.

### 4.5. In Vitro Release Studies

In vitro release experiments were conducted under sink conditions at 37 °C using simulated gastric (SGF) and intestinal fluids (SIF), containing 0.5% Tween 20 to increase the aqueous solubility of the polyphenol. The studies were performed under agitation in a slide-A-Lyzer^®^ Dialysis cassette 10.000 MWCO (Thermo scientific, Rockford, IL, USA). The cassette was filled with 3 mg resveratrol loaded in casein nanoparticles, previously dispersed in 5 mL water. Then, the cassette was introduced in a vessel containing 500 mL SGF (pH 1.2: 37 °C) under magnetic agitation. After 2 h in SGF, the cassette was introduced in another vessel containing 500 mL SIF (pH 6.8; 37 °C, under agitation). At different time points, samples tubes were collected and filtered with 0.45 µm filters (Thermo, Chino, CA, USA) before quantification.

The amount of resveratrol released from the formulations was quantified by HPLC. Calibration curves of free resveratrol in water containing 0.5% tween 20 at pH 1.2 and 6.8 were performed, over the range 0.05–6 µg/mL (*R*^2^ > 0.999) in both cases. To ascertain the drug release mechanism, the obtained data were fitted to the Korsmeyer-Peppas (Equation (2)) and the zero-order (Equation (3)) models.

M_t_/M_∞_ = K_KP_ × *t^n^* (2)

where M_t_/M_∞_ is the drug release fraction at time *t*, K_KP_ is a constant incorporating the structural and geometric characteristics of the matrix and n is the release exponent indicative of the drug release mechanism. The value of n indicates the mechanism of the release [45]. If the value is around 0.5, the mechanism is Case I (Fickian) diffusion, a value between 0.5 and 0.89 indicates anomalous (non-Fickian) diffusion suggesting a combination of mechanisms diffusion and erosion. Values of *n* between 0.89 and 1 indicate Case II transport, which involves a release mechanism ruled by erosion/relaxation of the matrix.

M_t_/M_∞_ = K_ZO_ × *t* (3)

where M_t_/M_∞_ is the drug release fraction at time *t*, and K_ZO_ is the zero-order release constant.

To fit the experimental data to the previous models, only the first portion of the release profile was used (M_t_/M_∞_ ≤ 0.6) [46].

### 4.6. Labelling of Casein Nanoparticles

#### 4.6.1. Radiolabeling of Casein Nanoparticles (NP-NOTA-Ga)

Casein nanoparticles were radiolabeled with ^67^Gallium (^67^Ga). Briefly, casein molecules were first tagged with p-SCN-Bn-NOTA (NOTA). Thus, 50 mg sodium caseinate and 5 mg NOTA were incubated for 24 h in 5 mL sodium bicarbonate solution (pH 9.5). After this reaction, the protein was purified by dialysis to remove the inorganic salts presented in the buffered medium and the free NOTA chelator. After this purification process, the protein derivative (casein-NOTA) was lyophilized. Tagged (50 mg) and untagged protein (450 mg) were used to prepare casein nanoparticles following the same procedure described above (see Section 4.2). Finally, NOTA nanoparticles (NOTA-NP) were labelled by citrate-NOTA transquelation with gallium-67 citrate [47].

#### 4.6.2. Fluorescently Labelling of Casein Nanoparticles (LR-NP)

Lumogen red-loaded nanoparticles were prepared by adding 2.5 mg Lumogen^®^ F Red 305 in acetone (5 mL) to the solution of sodium caseinate and lysine. Then, casein nanoparticles were formed by the addition of a calcium chloride water solution. The resulting nanoparticles were purified and dried in the Spray Drier apparatus under the same conditions described above (see Section 4.2).

### 4.7. In Vivo Biodistribution Studies

Studies were carried out in male Wistar rats (200–250 g) obtained from Harlan (Barcelona, Spain) and the protocols were approved by the Ethical Committee for Animal Experimentation of the University of Navarra (protocol numbers 117-12 [31 October 2012] and 059-13 [21 June 2013]). Prior to the experiments, animals were placed in metabolic cages and drink was provided *ad libitum*.

For radiolabeled nanoparticles, animals received a single dose of nanoparticles (10 mg gallium-67-citrate-NP). Animals were anesthetized with isofluorane and placed in prone position on the gamma camera. The SPECT-CT images were obtained 2 and 24 h after the administration of the radiolabeled nanoparticles in a Symbia^®^ gamma camera (Siemens Medical Systems, Malvern, PA, USA).

For fluorescently labelled nanoparticles, 30 mg nanoparticles in 1 mL water were administered to animals. Two hours later, the animals were sacrificed, and their gastrointestinal tract was removed. Jejunum portions of 1 cm were stored in the tissue proceeding medium OCT and frozen at −80 °C. Each portion was then cut into 5-µm sections on a cryostat and attached to glass slides. Finally, the samples were fixed with formaldehyde and incubated with DAPI for 15 min. The samples were examined in a fluorescence microscope (Axioimager M1, Zeiss, Oberkochem, Germany) with a coupled camera (Axiocam ICc3, Zeiss) and fluorescent source (HBO 100, Zeiss).

### 4.8. In Vivo Pharmacokinetic Studies in Wistar Rats

Pharmacokinetic studies were performed in male Wistar rats (200–250 g) obtained from Harlan (Barcelona, Spain). Studies were approved by the Ethical Committee for Animal Experimentation of the University of Navarra (protocol number 014-10 [12 February 2010]) in accordance with the European legislation on animal experiments. Before administration, animals were fasted overnight allowing free access to water.

For the pharmacokinetic study, rats were randomly divided into 4 groups (*n* = 6). The experimental groups that received the resveratrol formulations orally were: (i) resveratrol aqueous suspension (Rsv-Susp); (ii) resveratrol solution in PEG 400:water (Rsv-Sol) and (iii) resveratrol-loaded casein nanoparticles (Rsv-NP-C). As control, a group of animals received the resveratrol solution intravenously. Each animal received the amount of resveratrol equivalent to a dose of 15 mg/kg bw either orally or intravenously via tail vein.

Blood samples were collected at set times after administration (0, 10 min, 30 min, 1, 2, 4, 6, 8, 24 and 48 h) in specific plasma tubes (Microvette^®^ 500K3E, ref 20.1341. SARSTEDT, Nümbrecht Germany). Blood volume was recovered intraperitoneally with an equal volume of normal saline solution pre-heated at body temperature. Samples were immediately centrifuged at 10,000 rpm for 10 min. Plasma was separated into clean tubes and kept frozen at −80 °C until analysis of both resveratrol and resveratrol-3-*O*-d-glucuronide.

#### 4.8.1. Determination of Resveratrol and Rsv-3-*O*-d-Glucuronide Plasma Concentration by HPLC

The amount of resveratrol was determined by high pressure liquid chromatography with UV detection (HPLC-UV), following an analytical method previously published with minor modifications [48]. Analysis were carried out in an Agilent model 1100 series LC and diode-array detector set at 306 nm. The data were analyzed Chemstation G2171 program (B.01.03). The chromatographic system was equipped with a reversed-phase 250 mm × 2.1 mm C18 Kromasil (particle size 5 µm) column and a Gemini C18 support AJO-7596 precolumn. The mobile phase, pumped at 0.5 mL/min, was a mixture of water, methanol and acetic acid (50:45:5 by vol.) in isocratic conditions. The column was placed at 30 °C and the injection volume was 30 µL. Under these conditions, the run time for resveratrol-3-*O*-d-glucuronide and resveratrol was 6.2 ± 0.5 min and 12.6 ± 0.5 min, respectively.

For analysis, an aliquot of plasma (100 µL) was mixed with 50 µL HCl (0.1 N) and 500 µL acetonitrile to precipitate proteins. The mixture was shaken vigorously at 2500 rpm (Hettichzentrifugen, Tuttlingen, Germany) for 10 min. Then, the samples were centrifuged at 4000 rpm for 10 min and the supernatant was recovered and evaporated to dryness at 25 °C for 30 min in an Automatic environmental Speed vac^®^ system (Savant apparatus, Holbrook, NY, USA). Finally, 100 µL of an acetonitrile water mixture (1:1 by vol.) were added to reconstitute the extract. The resulting solution was filtered with 0.45 µm filters (Thermo, Chino, CA, USA) and injected in the HPLC system.

For quantification, calibration curves were designed over the range between 2 and 70 µg/mL, for the metabolite, and between 50 and 3000 ng/mL for resveratrol (*R*^2^ > 0.99). The standards were prepared by adding (resveratrol or resveratrol-3-*O*-d-glucuronide) in 500 µL acetonitrile to 100 µL free plasma following the same extraction method described above.

Under these experimental conditions, the limit of quantification for resveratrol was calculated to be 70 ng/mL. For the metabolite, the limit of quantitation of this technique was established as 4 µg/mL. Linearity, accuracy and precision values during the same day (intraday assay) at low, medium and high concentrations of resveratrol and resveratrol-3-*O*-d-glucuronide were always within the acceptable limits (less than 15%).

#### 4.8.2. Pharmacokinetic Data Analysis

Pharmacokinetic analysis was performed using a non-compartmental model with the WinNonlin 5.2 software (Pharsight Corporation, Cary, NC, USA). The following parameters were estimated: maximal serum concentration (C_max_), time in which C_max_ is reached (T_max_), area under the concentration-time curve from time 0 to the last time analyzed (AUC), mean residence time (MRT), clearance (Cl), volume of distribution (V) and half-life in the terminal phase (t_1/2_). Furthermore, the relative oral bioavailability (*Fr* %) of resveratrol was estimated by the following equation:

(4) $$Fr\;\left(\%\right)=\left(\frac{{AUC}_{oral}}{{AUC}_{iv}}\right)\times 100$$

where *AUC_i.v_*_._ and *AUC_oral_* were the areas under the curve for the iv and oral administrations, respectively.

### 4.9. In Vitro-In Vivo Correlations (IVIVC)

IVIVC were investigated by plotting a point-to-point between the amount of resveratrol released (FRD) from nanoparticles vs. the fraction of resveratrol absorbed (*FRA*) calculated from the mean plasma concentration-time inputs using the Wagner-Nelson equation [49]:

(5) $$FRA=\frac{C_{t}+k\times AUC_{0-t}}{k\times AUC_{0-\infty }}$$

where *C_t_* is the plasma concentration at a time *t*, *k* is the elimination rate constant, *AUC*_0–*t*_ is the area under the curve of the resveratrol plasma levels from 0 to time *t*, and *AUC*_0–∞_ is the area under the curve from 0 to infinity.

Then, linear regression analysis was applied and the coefficient of determination (R2) calculated.

### 4.10. Statistical Analysis

Data are expressed as the mean ± standard deviation (SD). The non-parametric Kruskall-Wallis followed by Mann-Whitney U-test was employed to investigate statistical differences. *p* < 0.05 was considered to be statistically significant. All data processing was performed using Graph Pad^®^ Prism statistical software.

## 5. Conclusions

Resveratrol can be successfully encapsulated into casein nanoparticles. These carriers displayed interesting properties for the oral administration of this polyphenol. First, casein nanoparticles released resveratrol following a zero-order kinetic and the release rate was not affected by pH conditions. Second, casein nanoparticles promoted the absorption of resveratrol offering sustained levels of the polyphenol in plasma for at least 8 h. Consequently, the oral bioavailability of resveratrol when encapsulated in casein nanoparticles was calculated to be close to 26.5%. Finally, a level IVIVC was found between in the amount of resveratrol released from nanoparticles and the percentage orally absorbed. This fact would be related with the capability of casein nanoparticles to both reach the surface of the gut epithelium and control the release rate of resveratrol, promoting its absorption and oral bioavailability.

## Figures and Tables

**Table 1:** Physicochemical characteristics of nanoparticles employed in this study.

Formulation	Size (nm)	PDI (nm)	Zeta Potential (mV)	Rsv Loading (µg/mg)	E.E. (%)
NP-C	138 ± 6	0.19 ± 0.02	−12 ± 1		
Rsv-NP-C	210 ± 3	0.20 ± 0.02	−19 ± 1	31 ± 1	68 ± 3

NP-C: empty nanoparticles; Rsv-NP-C: resveratrol-loaded casein nanoparticles. Data expressed as mean ± SD, *n* = 5.

**Table 2:** Pharmacokinetic parameters of resveratrol formulated as solution, after either intravenous or oral administration (Rsv-IV and Rsv-Sol, respectively), oral suspension (Rsv-Susp) or loaded in casein nanoparticles (Rsv-NP-C) at a single dose of 15 mg/kg bw to Wistar rats.

Formulation	Route	C_max_ (µg/mL)	T_max_ (h)	AUC (µg h/mL)	t_1/2_ (h)	Cl (mL/h)	Vd (mL)	MRT (h)	Fr (%)
Rsv-IV	iv	15.2 ± 5.18	0.1 ± 0.0	10.4 ± 3.80	2.0 ± 0.5	199.4 ± 89.81	569.2 ± 221.4	2.4 ± 1.0	100
Rsv-Sol	po	0.20 ± 0.02 **	0.6 ± 0.2	0.28 ± 0.13	0.3 ± 0.2 **	386.7 ± 224.9	112.3 ±103.6 **	1.3 ± 0.8 *	2.6
Rsv-Susp	po	-	-	-	-	-	-	-	-
Rsv-NP-C	po	0.29 ± 0.07 **^†^	1.8 ± 1.3	2.76 ± 1.64 **^††^	2.7 ± 0.7 **^††^**	161.8 ± 69.86	661.7 ± 242.9 **^††^**	8.2 ± 3.7 **^††^	26.5

Data are expressed as mean ± SD (*n* = 6). C_max_: peak plasma concentration; T_max_: time to reach plasma concentration; AUC: Area under the curve; t_1/2_: half-life of the terminal phase; Cl: Clearance; MRT: mean residence time Fr: relative oral bioavailability. * Significant differences vs. Rsv-IV (*p* < 0.05) (Mann-Whitney-U); ** Significant differences vs. Rsv-IV (*p* < 0.01) (Mann-Whitney-U); **^†^** Significant differences vs. Rsv-Sol (*p* < 0.05) (Mann-Whitney-U); **^††^** Significant differences vs. Rsv-Sol (*p* < 0.01) (Mann-Whitney-U).

## Abbreviations

AUC	Area under the curve
Cl	Clearance
C_max_	peak plasma concentration
DAPI	4′,6-diamidino-2-phenylindole
E.E.	encapsulation efficiency
Fr	relative oral bioavailability
FRA	fraction of resveratrol absorbed
FRD	fraction of resveratrol released
IVIVC	In vitro-in vivo correlations
LR-NP	Lumogen red-loaded casein nanoparticles
MRT	mean residence time
NP-C	empty casein nanoparticles
NP-NOTA-Ga	casein nanoparticles radiolabeled with Gallium-67
PCS	photon correlation spectroscopy
PDI	polydispersity index
PEG	polyethylene glycol 400
Rsv	resveratrol
Rsv-IV	resveratrol solution intravenously administered
Rsv-NP-C	resveratrol-loaded casein nanoparticles
Rsv-Sol	solution of resveratrol in a mixture of PEG 400 and water
Rsv-Susp	aqueous suspension of resveratrol
SGF	simulated gastric fluid
SIF	simulated intestinal fluid
t_1/2_	half-life of the terminal phase
T_max_	time to reach plasma concentration
